# Raspberry‐Like Microspheres of Core–Shell Cr_2_O_3_@TiO_2_ Nanoparticles for CO_2_ Photoreduction

**DOI:** 10.1002/cssc.201901712

**Published:** 2019-10-17

**Authors:** Jeannie Z. Y. Tan, Fang Xia, M. Mercedes Maroto‐Valer

**Affiliations:** ^1^ Research Centre for Carbon Solutions (RCCS) Heriot–Watt University Edinburgh EH14 4AS UK; ^2^ Discipline of Chemistry and Physics, College of Science, Health, Engineering, and Education Murdoch University Murdoch Western Australia 6150 Australia

**Keywords:** CO_2_ conversion, core–shell nanoparticles, photocatalysis, solar fuels, X-ray diffraction

## Abstract

To promote the interaction of p–n semiconductors, raspberry‐like microspheres of core–shell Cr_2_O_3_@TiO_2_ nanoparticles have been fabricated through a five‐step process. Raman spectroscopy of products calcined at various temperatures reveal that the titania shell causes crystal distortion of the Cr_2_O_3_ core, without changing the microstructures of the fabricated core–shell microspheres. In situ and time‐resolved synchrotron‐based powder XRD reveals the formation of monoclinic TiO_2_ in the fourth step, but these monoclinic TiO_2_ nanocrystals undergo a phase transition when the applied calcination temperature is above 550 °C. As a result, TiO_2_(B), a magnéli phase of Ti_4_O_7_ and Cr_2_Ti_6_O_15_ compounds, resulting from inner doping between Cr_2_O_3_ and TiO_2_, is formed. The close interaction of Cr_2_O_3_ and TiO_2_ forms a p–n junction that decreases the recombination of photogenerated electron–hole pairs, leading to enhanced production of CH_4_ by photocatalytic reduction of CO_2_.

## Graphical abstract

**Giving CO_2_ conversion the raspberry**: To promote the interaction of p–n semiconductors, raspberry‐like core–shell Cr_2_O_3_@TiO_2_ nanoparticles were fabricated through a five‐step process. The products calcined at different temperatures revealed that the titania shell resulted in the crystal distortion of the Cr_2_O_3_ core, without changing the microstructures of the fabricated core–shell microspheres.

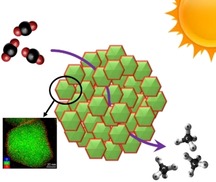

## Introduction

In contrast to the use of fossil fuels and associated adverse global environmental effects, solar energy has the potential to provide our energy demands if it can be efficiently harvested and transformed. The photocatalytic reduction of CO_2_ with H_2_O to valuable hydrocarbons, such as methane or methanol, is promising to reduce CO_2_ emissions, as well as offering renewable energy alternatives.^1^ However, the efficiency of CO_2_ photoreduction is still very low to date. To improve the conversion efficiency, the development of nanomaterials with well‐defined sizes, shapes, crystal phases, structure and composition are becoming increasingly important.^2^

Nanomaterials have attracted enormous attention owing to their interesting properties and applications in diverse areas, such as photocatalysis,^3^ nanoelectronics,^4^ and integrated catalysis.^5^ The preparation of such materials is currently regarded as among the most challenging areas in chemistry.^2^ In particular, core–shell nanostructures with conducive and versatile compositions and structures are highly desirable in certain applications as they are task‐specific nanomaterials with multifunctional capabilities.^2^, ^6^ Moreover, nanomaterials with synergetic properties between the core and the shell have become a very important class for emerging applications, such as enhanced optical devices,^7^ tailored magnetic materials,^8^ energy storage materials,^9^ fuel cells,^10^ dye‐sensitized solar cells^11^ and many important catalytic^12^ and photocatalytic reactions.3b, 13

Chromium(III) oxide (Cr_2_O_3_, eskolaite), which is a p‐type semiconductor with a band gap of approximately 3.5 eV, has been recently proposed and used as a photocatalyst or co‐catalyst for different photocatalytic reactions.3a, 14 Maeda et al. proposed that the oxidation and reduction reactions in the photocatalytic overall water splitting process could be separated by using core–shell Rh/Cr_2_O_3_ nanostructures.3a To overcome the rapid recombination of photogenerated electrons and holes, Hu et al. proposed the coupling between Cr_2_O_3_ and WO_3_ to induce the formation of p–n junctions.14a Chen et al. suggested that Cr_2_O_3_ could act as the surface holes trapper within the Cr_2_O_3_–carbon nanotubes/TiO_2_ nanocomposite to reduce the recombination rate of the photogenerated electron–hole pairs.^15^ A recent study proposed that the presence of oxygen vacancies within Cr_2_O_3_ could be used for methanol synthesis.^16^

The fabrication of the core–shell structured Cr_2_O_3_:P@fibrous‐phosphorus hybrid composites revealed that the core–shell structure not only can reduce the recombination of the photogenerated electron–hole pairs, but also can enhance the optical properties of the composite.3b Doping of P into Cr_2_O_3_ at the interface of the fibrous‐phosphorus and Cr_2_O_3_ composite resulted in the extension of the absorption tail into the near IR region, which can neither be observed in Cr_2_O_3_ nor in fibrous‐phosphorus. Moreover, Cao et al. reported that the inner doping of Cr on TiO_2_ thin films could significantly enhance the photo(electro)catalytic water splitting efficiency.^17^ Recently, Zhao and co‐workers proposed that the use of core–shell structures offers an excellent system for light/chemical CO_2_ photoreduction. For instance, the wrapping of reduced graphene oxide onto the Pt‐decorated thin sheet TiO_2_ exhibited an apparent quantum efficiency of 1.93 % in the CO_2_ photoreduction reaction to CH_4_.^18^ The group also proposed the fabrication of core–shell bimetallic (i.e., Au@Pd and Pt@Ru) nanoparticles decorated on TiO_2_ to enhance the optical properties of TiO_2_, reduce the recombination of photogenerated charges and improve the CO_2_ adsorption capability for CO_2_ photoreduction.^19^ In addition, the product selectivity of CO_2_ photoreduction can be tuned through adjusting the Au/Pd molar ratio on TiO_2_.19b

In this work, we aim at achieving p–n nanojunctions between Cr_2_O_3_ and TiO_2_ to enhance the interaction between the p‐ and n‐type semiconductors, and consequently, increase CO_2_ photoreduction efficiency. Unlike the typical core–shell structure, which consists of a layer of shell coated on a core particle, novel raspberry‐like microspheres were constructed herein from many core–shell Cr_2_O_3_@TiO_2_ nanoparticles. A thin layer of TiO_2_ was coated on the Cr_2_O_3_ nanoparticles of the microsphere, establishing the p–n nanojunction between Cr_2_O_3_ and TiO_2_. The microstructure and elemental analysis of the nanocomposite were examined to characterize the morphology and distribution of TiO_2_ on the Cr_2_O_3_. Using in situ and time‐resolved synchrotron‐based powder X‐ray diffraction (PXRD) and Raman spectroscopy, the change of crystal phase of the fabricated Cr_2_O_3_@TiO_2_ nanocomposite with increasing calcination temperature was observed. The photocatalytic efficiencies of the pristine Cr_2_O_3_ and the fabricated Cr_2_O_3_@TiO_2_ were evaluated for CO_2_ photoreduction.

## Results and Discussion

**Figure 1:**
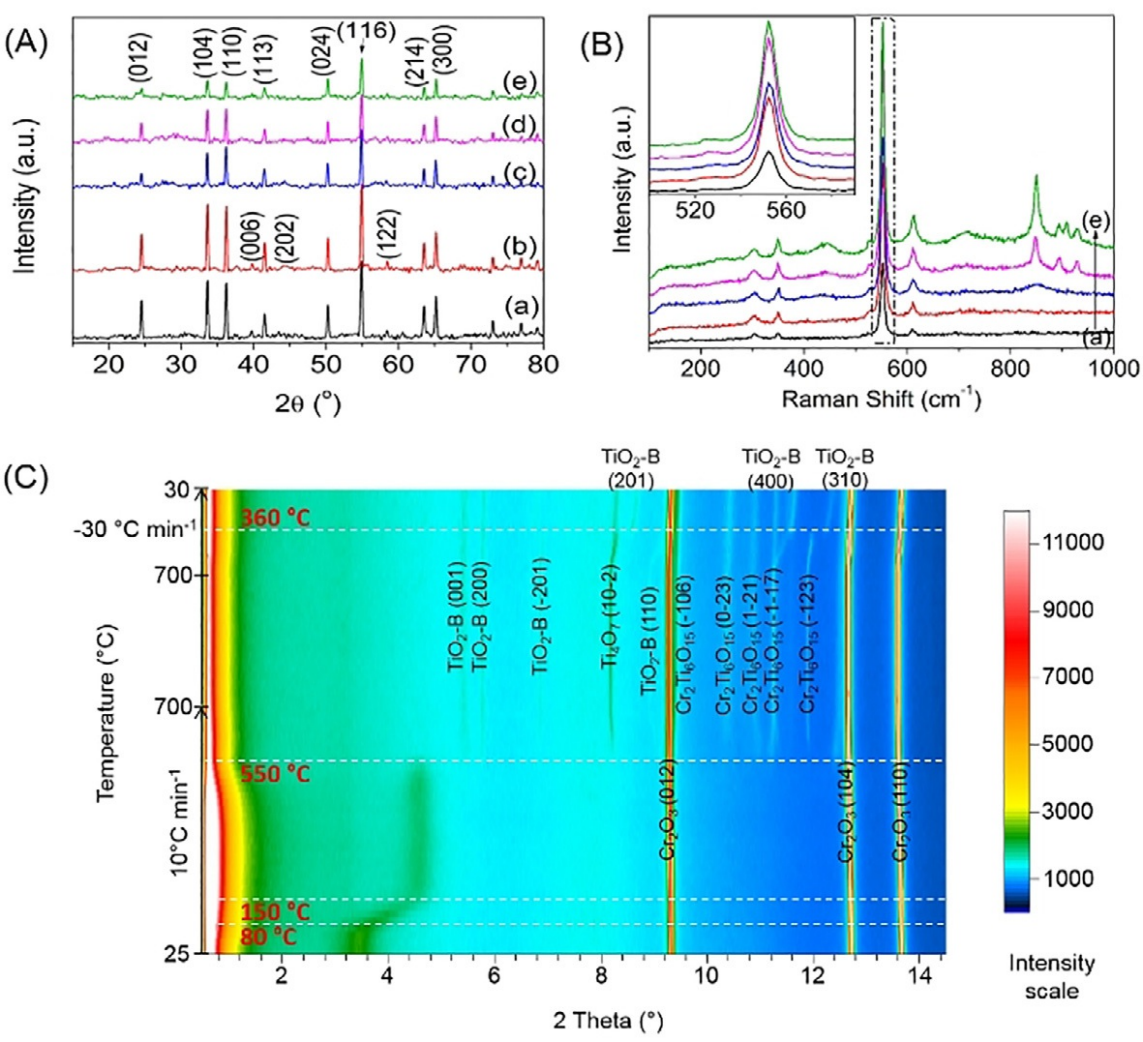
Laboratory‐based PXRD pattern (A) and Raman spectra (B) of as‐prepared Cr_2_O_3_ (a), 400‐Cr_2_O_3_@TiO_2_ (b), 550‐Cr_2_O_3_@TiO_2_ (c), 700‐Cr_2_O_3_@TiO_2_ (d), 850‐Cr_2_O_3_@TiO_2_ (e). In situ and time‐resolved synchrotron‐based PXRD diagram of Cr_2_O_3_@TiO_2_ after the alkaline hydrothermal etching assisted crystallization method (C).

The laboratory and synchrotron‐based PXRD patterns of the as‐prepared Cr_2_O_3_ revealed high crystallinity and the peaks matched well with Cr_2_O_3_ in the database (eskolaite, JCPDS No.: 38‐1479; Figure 1 A,C). The laboratory‐based diffraction patterns showed that the Cr_2_O_3_ phase in the mixture had no observable changes in peak positions after calcination at all temperatures (Figure 1 A). The absence of the TiO_2_ peak was probably due to the low amount of TiO_2_ within the sample. When the calcination temperature increased from 400 to 850 °C, all the peaks of Cr_2_O_3_ remained (Figure 1 A). However, the overall peak intensity of the laboratory‐based PXRD spectra decreased, indicating a possible phase transition triggered in Cr_2_O_3_ during the calcination treatment at different temperatures (this is discussed further below; Figure 1 A, c–e).^20^ Moreover, a number of new peaks between 24.5–33.3° were observed in the laboratory‐based PXRD. To study the effect of calcination and to elucidate the new peaks observed in the laboratory‐based PXRD, Raman spectroscopy was carried out (Figure 1 B) and in situ and time‐resolved synchrotron‐based PXRD were conducted (Figure 1 C).

Raman spectroscopy is a powerful technique to probe the crystal lattice vibrations in the study of nanomaterials.^21^ When the calcination temperature was increased to 550 °C, new weak peaks centered at approximately 113.4 and 437.9 cm^−1^, which were assigned to TiO_2_(B), appeared (Figure 1 B).^22^ Moreover, the shoulder peak at approximately 417 cm^−1^ was very likely a result of magnéli Ti_4_O_7_.^23^ The peaks centered at 302.6, 349.2, 523.6, 551.2 and 611.3 cm^−1^ can be assigned to crystalline Cr_2_O_3_ (Figure 1 B).^24^ The pattern of these peaks did not change with increasing calcination temperatures, but the intensities did. The Raman intensity of the Cr_2_O_3_@TiO_2_ nanocomposites increased with calcination temperature compared with the pristine Cr_2_O_3_ (inset of Figure 1 B). Raman intensity has been used to investigate alterations of crystal structure, evaluating the distortions of the crystal structure.^25^ It is proposed here that the host lattice, which was the core Cr_2_O_3_, was distorted by the shell titania during the calcination treatment. With the increase in calcination temperature, the extent of the distortion was enhanced, resulting a stronger intensity of the Raman spectra. Moreover, owing to the reorientation of Cr_2_O_3_ crystals, new peaks centered at 446.2, 713.5, 847.6, 906.7 and 927.6 cm^−1^ emerged. However, this crystal reorientation or distortion would cause the decrease in crystallinity of Cr_2_O_3_. Hence, the laboratory‐based PXRD intensity of the TiO_2_/Cr_2_O_3_ nanocomposites decreased with the calcination temperature (Figure 1 A).

Throughout the in situ PXRD experiment, the strong characteristic peaks of Cr_2_O_3_ at 9.3°, 12.7° and 13.7° were indexed to the (0 1 2), (1 0 4), and (1 1 0) lattice planes, respectively (Figure 1 C). In addition, there was a broad peak centered at approximately 3.4°, which was later shifted to approximately 4.6° when the temperature was elevated from 80 to 550 °C, corresponding to the monoclinic TiO_2_ (JCPDS No.: 65‐6429), which was observed previously in a TiO_2_ nanocomposite calcined below 550 °C.^26^ The shift of this broad peak during the in situ calcination process could be due to the thermal contraction of TiO_2_. When the in situ calcination temperature was further increased to 700 °C, this broad peak disappeared because the monoclinic TiO_2_ was unstable above 550 °C. Beyond 550 °C, several new peaks appeared and they were assigned to TiO_2_(B) (JCPDS No.: 46‐1237), magnéli phase Ti_4_O_7_ (JCPDS No.: 50–0787) and Cr_2_Ti_6_O_15_ (JCPDS No.: 30‐0419) as indicated in Figure 1 C. These newly emerged phases were not detected in the laboratory‐based PXRD pattern (Figure 1 A) probably because the amount of these new phases was too low to be detected by using the laboratory‐based PXRD. This newly formed Cr_2_Ti_6_O_15_ phase was also confirmed in the Raman spectra (multiple peaks positioned at 719.2 and 851.3 cm^−1^; Figure 1 B, d and e),^27^ further confirming the presence of Cr_2_Ti_6_O_15_ in the samples calcined at 550–850 °C.

**Figure 2:**
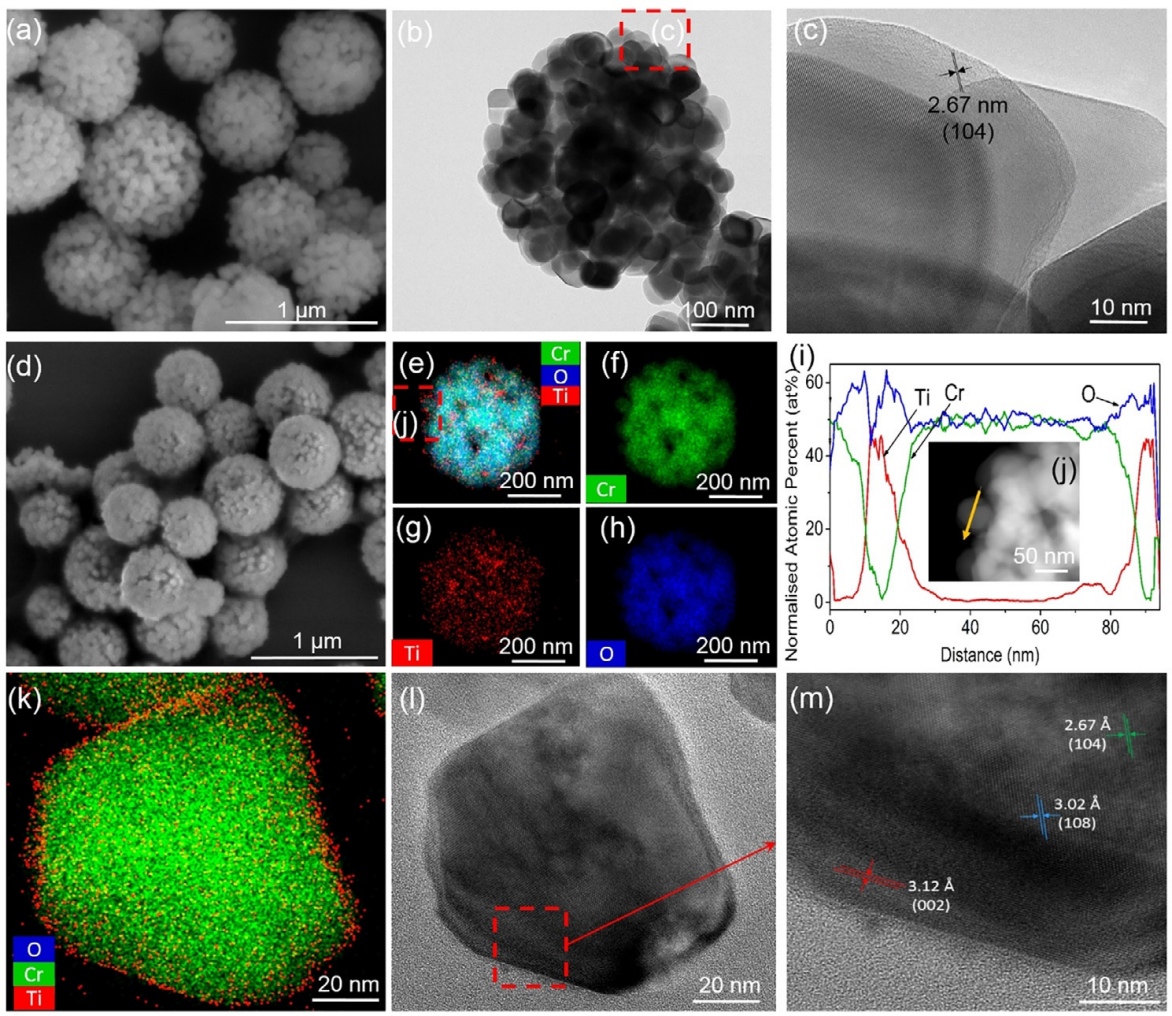
SEM (a), TEM (b) and HRTEM (c) images of Cr_2_O_3_ before incorporating TiO_2_. SEM (e) and TEM‐EDX elemental mapping diagram (e–h) of 700‐Cr_2_O_3_@TiO_2_. Elemental line scan profile (i) of one of the nanoparticles within a 700‐Cr_2_O_3_@TiO_2_ microsphere (j). High‐resolution TEM‐EDX (k) and TEM images (l and m).

The pristine Cr_2_O_3_ microspheres exhibited a raspberry‐like microstructure, which is composed of many nanoparticles (Figure 2 a,b). The measured lattice fringes were 0.27 nm, which is in agreement with the (1 0 4) lattice plane of Cr_2_O_3_ (Figure 2 c). After the coating of SiO_2_ and titanium(IV) butoxide (TBT), the surface of the microspheres became smoother, losing the raspberry‐like microstructure (see the Supporting Information, Figure S1 a, b). Then, the SiO_2_ protective layer was removed by using an alkaline hydrothermal etching assisted crystallization method, which also crystallized the titania layer, although the crystallinity was very weak (Figure 1 C starting from room temperature). Excessive TBT coated on the SiO_2_ layer was also removed together with SiO_2_ (Figure S1 b). As a result, the Cr_2_O_3_@TiO_2_ microspheres recovered the raspberry‐like microstructure (Figure 2 d). No significant change in the overall spherical morphology was observed after calcination. After the removal of SiO_2_ and calcination treatment at 700 °C, the Cr_2_O_3_@TiO_2_ sample exhibited highly dispersed and homogeneous distribution of titania within each microsphere (Figure 2 e–h). The TEM‐EDX (energy‐dispersive X‐ray) line scan profile revealed that the thickness of Ti was about 10 nm, coating on Cr_2_O_3_ nanoparticle (Figure 2 i), which was also observed by TEM (Figure S2). The successive coating of titania on each of the Cr_2_O_3_ nanoparticles was promoted by the porous structure of the Cr_2_O_3_ core (Figure 2 j).

After the removal of SiO_2_, the close contact of the core and shell layers (Figure 2 k,l), which were the Cr_2_O_3_ and TiO_2_, respectively, formed the oxide composite (i.e., Cr_2_Ti_6_O_15_) as revealed by the in situ PXRD (Figure 1 C). This was further evident from the HRTEM, in which the lattice spacing of the Cr_2_Ti_6_O_15_ (1 0 8) facet with 3.02 Å, was observed in between the TiO_2_ (0 0 2) and Cr_2_O_3_ (1 0 4) facets (Figure 2 m).

**Figure 3:**
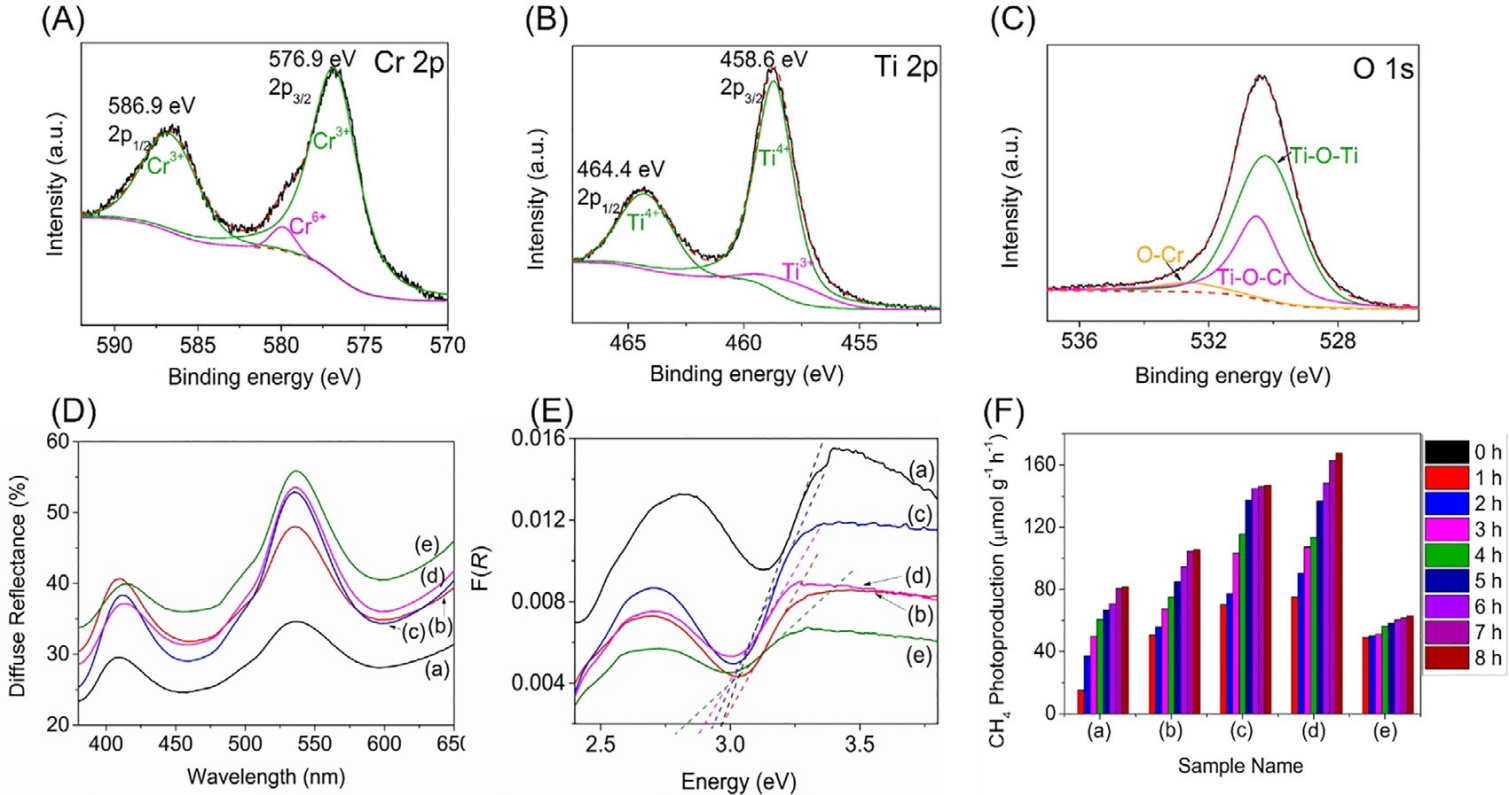
High‐resolution XPS spectra of Cr 2p (A), Ti 2p (B), O 1s (C) for 700‐Cr_2_O_3_@TiO_2_ sample. Diffuse reflectance (D), Kubelka–Munk function (E) and cumulative CH_4_ photoproduction (F) of the as‐prepared Cr_2_O_3_ (a), 400‐Cr_2_O_3_@TiO_2_ (b), 550‐Cr_2_O_3_@TiO_2_ (c), 700‐Cr_2_O_3_@TiO_2_ (d), 850‐Cr_2_O_3_@TiO_2/_ (e).

The surface chemistry of 700‐Cr_2_O_3_@TiO_2_ was elucidated by using X‐ray photoelectron spectroscopy (XPS). High‐resolution Cr 2p spectra (Figure 3 A) exhibited two major components centered at 576.9 and 586.9 eV attributed to Cr^3+^ species from Cr_2_O_3_.^28^ Minor components positioned at 579.9 and 589.1 eV corresponded to the Cr^6+^ from the chromium precursor.^29^ The high‐resolution Ti 2p (Figure 3 B) spectrum presented two major components at 458.6 and 464.4 eV, which are assignable to Ti^4+^.^9^ The presence of Ti^3+^ from Ti_4_O_7_ was exhibited in the small peak centered at 457.7 eV.^9^ The O 1s spectra were numerically fitted with three types of surface oxygen centered at 530.3, 530.5 and 532.6 eV, which were assigned to Ti‐O‐Ti, Ti‐O‐Cr and O‐Cr, respectively (Figure 3 C).^9^, ^28^

The as‐prepared Cr_2_O_3_ sample exhibited two absorption peaks at approximately 400 and 535 nm (Figure S3), originating from the ^4^A_2g_→^4^T_1g_ and ^4^A_2g_→^4^T_2g_ electronic transitions in Cr_2_O_3_, respectively.^30^ The former is characteristic of the Cr^3+^ ions of six‐coordinate geometry and the latter implies octahedral symmetry. A dramatic decline in the absorption intensity, particularly for the trough centered at about 535 nm (Figure S3), in the fabricated Cr_2_O_3_@TiO_2_ samples was observed when the calcination temperature was increased from 400 to 850 °C. This phenomenon was very likely due to the distortion of the octahedral symmetry in the Cr_2_O_3_ by the titania moiety, as evident from the formation of Cr_2_Ti_6_O_15_ (Figure 1 C), during the calcination process.^31^ The distortion of the octahedral symmetry slightly decreased the band gap energy of the fabricated Cr_2_O_3_@TiO_2_ nanocomposites, which was derived from the diffuse reflectance spectra (Figure 3 D), from 3.0 to 2.8 eV (Figure 3 E). Although this variation in electronic property has been reported in the literature previously, the mechanism is still unclear and further studies are needed.^31^

The photoreduction of CO_2_ was conducted for 8 h for the Cr_2_O_3_ sample and the calcined Cr_2_O_3_@TiO_2_ samples (Figure 3 F). The photoproducts were analyzed after each hour. In the control experiments, no methane was produced in the absence of photocatalyst, water and CO_2_ under UV light irradiation. When the photocatalyst was loaded into the reactor in the dark (0 h), no product was obtained. When the reaction was run with He (i.e., presence of photocatalysts and water under UV light irradiation), no conversion was observed. However, in the presence of photocatalyst, CO_2_, H_2_O, and UV light, the production of CO in a trace amount and CH_4_ was observed (Table S1). The photoproduction of CH_4_ was genuine, which was evident from the negligible weight loss of 400‐Cr_2_O_3_@TiO_2_ in the thermogravimetric analysis (TGA) spectrum (Figure S4). The maximum cumulative photoproduction of CH_4_ from CO_2_ by the pristine Cr_2_O_3_ was approximately 82 μmol g_catalyst_ h^−1^ (Figure 3 F). The photoproduction of CH_4_ was significantly enhanced when the core–shell structured Cr_2_O_3_@TiO_2_ sample was used. The maximum cumulative photoproduction of CH_4_ increased with the calcination temperature of the Cr_2_O_3_@TiO_2_ samples, from approximately 105 μmol g_catalyst_ h^−1^ for sample 400‐Cr_2_O_3_@TiO_2_ to about 168 μmol g_catalyst_ h^−1^ for sample 700‐Cr_2_O_3_@TiO_2_ (AQE=0.296 %; Figure 3 F). Further increasing the calcination temperature to 850 °C had a detrimental effect on the photoreduction performance, leading to only about 63 μmol g_catalyst_ h^−1^ of cumulative CH_4_ production. This was probably because when the calcination temperature was increased to 850 °C, the inner doping between TiO_2_ and Cr_2_O_3_ was greatly enhanced, resulting in crystal distortion and reorientation in the Cr_2_O_3_ by the titania moiety, as observed in the Raman studies. As a result, the overall crystallinity of the 850‐Cr_2_O_3_@TiO_2_ sample was decreased, leading to an inferior photocatalytic activity. When using the optimized sample, 700‐Cr_2_O_3_@TiO_2_, the photoreduction of CO_2_ was further tested up to 16 h to investigate the durability of the photocatalyst. The cumulative production of CH_4_ was stabilized at 171.4 μmol g_catalyst_ h^−1^, indicating the photoproduction rate of CH_4_ decreases with time (Figure S5).

The increase in the CH_4_ production of the 400‐, 550‐ and 700‐Cr_2_O_3_@TiO_2_ samples compared with the pristine Cr_2_O_3_ was due to the core–shell microstructure, which resulted in a close contact of the p–n junction formed between Cr_2_O_3_ and TiO_2_, as seen in the TEM‐EDX line mapping. In addition, the inner doping at the interface between TiO_2_ and Cr_2_O_3_, which resulted in the formation of Cr_2_Ti_6_O_15_, was speculated to enhance the photocatalytic activity, which was also reported previously.^17^ The heterojunction formed thus increased the charge separation efficiency as evident from the photoluminescence spectra (PL; Figure S6). Based on the characterization results, the overall photocatalytic mechanism was proposed. Upon irradiation with UV light, electrons in TiO_2_ and Cr_2_O_3_ were excited from their respective valence bands to their conduction bands. The formation of p–n junctions in the core–shell microstructure shortened the migration distance of the photogenerated electrons. As a result, the photogenerated electrons in Cr_2_O_3_ migrated to the conduction band of TiO_2_; whereas the photogenerated holes accumulated at the valence band of Cr_2_O_3_.14b, 32 The electrons then photoreduced the adsorbed CO_2_ to CH_4_.^32^ Meanwhile, the accumulated photogenerated holes underwent photooxidation of H_2_O.

## Conclusions

Raspberry‐like structured Cr_2_O_3_@TiO_2_ microspheres were synthesized by using a five‐step process involving hydrothermal and calcination treatments. The pristine Cr_2_O_3_ microspheres were made up from many Cr_2_O_3_ nanoparticles, which were coated with a thin layer of TiO_2_ shell on each of the Cr_2_O_3_ nanoparticles, forming a novel core–shell nanostructure within a microsphere. Different calcination temperatures (i.e., 400–850 °C) were applied to the Cr_2_O_3_@TiO_2_ samples after the removal of the SiO_2_ layer. When the applied calcination temperature was above 550 °C, inner doping at the interface of Cr_2_O_3_ and TiO_2_ nanoparticles through the formation of Cr_2_Ti_6_O_15_ was observed in the Raman spectra and synchrotron‐based in situ PXRD. The p–n heterojunction between Cr_2_O_3_ and TiO_2_ at the nanoscale level and the newly formed Cr_2_Ti_6_O_15_ phase were proposed to enhance the CO_2_ photocatalytic reduction efficiency with a maximum cumulative product of approximately 168 μmol g_catalyst_^−1^ h^−1^ when the Cr_2_O_3_@TiO_2_ sample was calcined at 700 °C.

## Experimental Section

### Chemicals

Absolute ethanol (ACS reagent), K_2_Cr_2_O_7_ (≥99.0 %), acrylamide solution (40 % in H_2_O), tetraethyl orthosilicate (TEOS, 98 %), NH_3_ (28 wt %), titanium(IV) butoxide (TBT, 97 %), NaOH (pellets EMPLURA) and HCl (37 wt %) were all purchased from Sigma–Aldrich. All the reagents were used as received. Milli‐Q water (resistivity higher than 18.2 MΩ cm^−1^) was collected from a Millipore academic purification system.

### Method

**Scheme 1:**
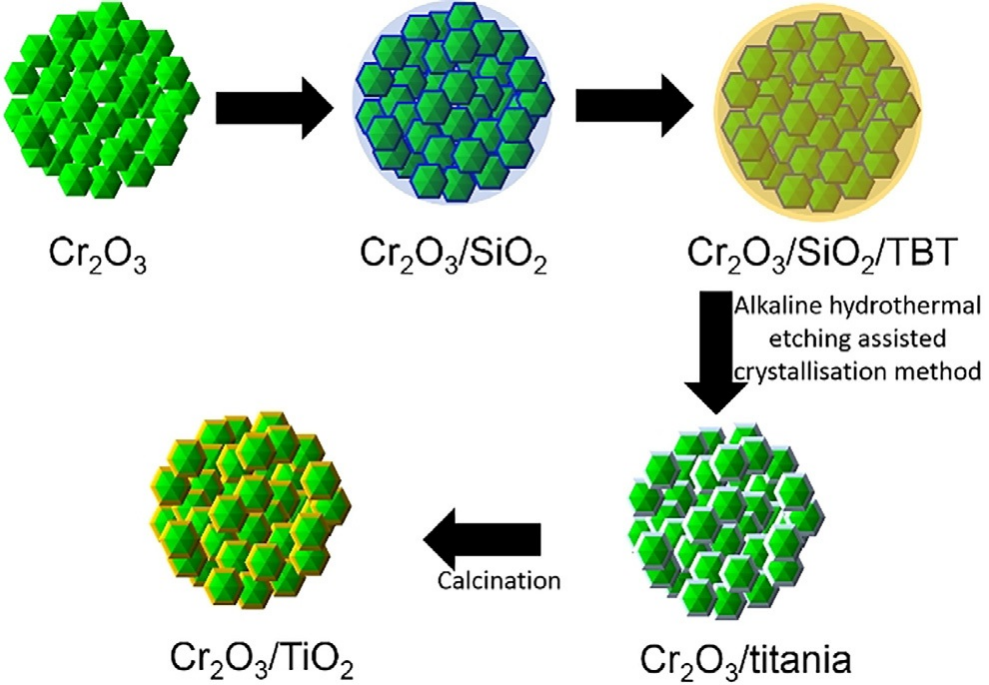
Schematic diagram of the five‐step synthesis process.

Raspberry‐like microspheres consisting of core–shell Cr_2_O_3_@TiO_2_ nanoparticles were fabricated by following a five‐step process (Scheme 1), as described below.

#### 1 Synthesis of Cr_2_O_3_ microspheres

The synthesis of Cr_2_O_3_ was adopted from a previous study.^33^ Briefly, K_2_Cr_2_O_7_ (0.5 mmol), acrylamide (1.0 mmol) and H_2_O (2.1 mol) were mixed and stirred until a homogeneous orange solution was obtained. The solution was then transferred into a 100 mL Teflon‐lined stainless‐steel autoclave and treated at 180 °C for 12 h. After the hydrothermal treatment, the obtained dark‐green powder was washed consecutively in a series of sequential washing steps alternating ethanol and water for 3 times. After drying the powder in an oven at 75 °C, the product was calcined at 900 °C with ramping 10 °C min^−1^ and maintained at 900 °C for 1 h.

#### 2 Synthesis of Cr_2_O_3_@SiO_2_ microspheres

The synthesis of Cr_2_O_3_@SiO_2_ microspheres was prepared according to a previous study.8b First, Cr_2_O_3_ microspheres (0.15 g) were dispersed in ethanol (3 mL). Second, the mixture was added into a round‐bottom flask containing ethanol (280 mL), Milli‐Q water (70 mL) and concentrated NH_3_ (5 mL, 28 wt %) in an ultrasound bath and treated ultrasonically for 30 min. After that, TEOS (4 mL) was added dropwise (flow rate: 0.4 mL min^−1^). Then, the solution was kept under continuous mechanical stirring for 12 h at room temperature. Finally, Cr_2_O_3_@SiO_2_ microspheres were separated by using a centrifuge (ThermoFisher Sarvall Primo Benchtop Centrifuge, 4000 rpm for 15 min), followed by washing consecutively in a series of sequential washing steps alternating ethanol and water for 3 times.

#### 3 Synthesis of Cr_2_O_3_@SiO_2_@TBT microspheres

The obtained product of Cr_2_O_3_@SiO_2_ microspheres was redispersed in ethanol (200 mL) and mixed with concentrated NH_3_ (0.9 mL, 28 wt %) under ultrasound for 30 min. After that, TBT (2 mL) was added dropwise (flow rate: 0.4 mL min^−1^). Then, the solution was kept under continuous mechanical stirring for 24 h at 45 °C. The resulting Cr_2_O_3_@SiO_2_@TBT microspheres were separated from the solution by using a centrifuge (4000 rpm for 15 min), followed by washing consecutively in a series of sequential washing steps alternating ethanol and water for 3 times.

#### 4 Synthesis of Cr_2_O_3_@titania microspheres

The removal of SiO_2_ was conducted by using an alkaline hydrothermal etching assisted crystallization method.8b The Cr_2_O_3_@SiO_2_@TBT product obtained in the previous step was mixed with NaOH solution (20 mL, 1.0 m). The solution was then transferred to a 100 mL Teflon‐lined stainless‐steel autoclave. The autoclave was heated at 150 °C for 24 h, and then allowed to cool down to room temperature in air. The product obtained was then immersed in aqueous HCl (100 mL, 0.1 m) for 20 min, and subsequently washed with Milli‐Q water until the pH value was close to 7, and then was dried at 75 °C in an oven overnight.

#### 5 Calcination of Cr_2_O_3_@titania microspheres

The dried product was calcined at 400, 550, 700 or 850 °C in air and the resultant samples were denoted as 400‐Cr_2_O_3_@TiO_2_, 550‐Cr_2_O_3_@TiO_2_, 700‐Cr_2_O_3_@TiO_2_ and 850‐Cr_2_O_3_@TiO_2_, respectively.

### Characterization

The morphology of the synthesized Cr_2_O_3_ and the Cr_2_O_3_@TiO_2_ samples after calcination at different temperatures was examined by field emission scanning electron microscopy (FE‐SEM, Quanta 200 F FEI), transmission electron microscopy (TEM) and high‐resolution (HR) TEM (FEI Titan Themis 200) operated at 200 kV. The TEM was equipped with an energy‐dispersive X‐ray spectroscopy (EDX) detector. To investigate the structures of the nanospheres, samples were embedded in TAAB 812 resin and sliced into approximately 90 nm thick sections. The crystallinity of the synthesized products was assessed by powder X‐ray diffraction (laboratory‐based PXRD, Bruker Advanced Diffractometer) equipped with Cu_Kα_ source (*λ*=1.5418 Å). In situ and time‐resolved synchrotron‐based PXRD studies were carried out at the Australian Synchrotron at the Powder Diffraction beamline. The X‐ray energy was 21 keV, and the wavelength (*λ*=0.590928 Å) was calibrated by using a LaB_6_ standard (NIST SRM 660b). The methodology was similar to previous in situ PXRD studies.^34^ The precursor sample (dried Cr_2_O_3_@TBT) was loaded into a quartz glass capillary (1.0 mm OD and 0.1 mm wall thickness); silica glass wool plugs were placed before and after the sample section to prevent sample movement during oscillation and heating. Both ends of the capillary were open to air. The loaded capillary was placed at the X‐ray beam center and heated (10 °C min^−1^) to the target temperature (700 °C) by a hot air blower under the capillary. The temperature was sensed by a K‐type thermocouple about 2 mm beneath the capillary and was calibrated by using KNO_3_ and quartz temperature standards. In situ PXRD patterns were collected during the calcination process by using a position‐sensitive MYTHEN detector over the 2*θ* range 1.5–81.5° with a time resolution of 2 min. Raman spectra were collected by using a Renishaw inVia Raman Microscope with 514 nm excitation source. X‐ray photoelectron spectroscopy (XPS) was performed with a Scienta 300 XPS machine incorporated with a rotating Al_Kα_ X‐ray source operating at 13 kV, 333 mA (4.33 kW). Electron analysis was performed by using a 300 cm radius hemispherical analyzer and lens system. The electron counting system consists of a multichannel plate, phosphorescent screen and CCD camera. All multichannel detection counting is done by using proprietary Scienta software. The elements present were determined by a wide energy range survey scan (200 mV step, 20 ms dwell time, 150 eV pass energy and summed over three scans). The high‐resolution scans were performed at a similar pass energy (150 eV) as in the survey scan, but a step size of 20 mV, a dwell time of 533 ms and summed over three scans. The instrument operated at a base pressure of 1×10^−9^ mbar; the energy scale is calibrated by using the Au 4f, Ag 3d and Cu 2p emission lines, where the half width of the Au 4f_7_ emission line is approximately 1.0 eV. All of the sample was mixed with a small amount of Ag powder to act as a binding energy reference. All data analysis and peak fittings were performed by using the CasaXPS software. Diffuse reflectance studies were carried out by using a UV/Vis spectrometer (PerkinElmer Lambda 900) equipped with an integrating sphere (150 mm). Sample weight loss was analyzed by using a TGA Q500 Thermogravimetric Analyzer from TA Instruments in the air (ramping temperature 10 °C min^−1^ up to 900 °C). Photoluminescence (PL) was performed with a Fluorescence Spectrometer (PerkinElmer LS 55) with a 300 nm excitation wavelength and a cut‐off filter at 350 nm.

### Photocatalytic testing

The prepared samples were tested for CO_2_ photoreduction, as described previously.^35^ Briefly, Cr_2_O_3_@TiO_2_ powder (0.1 g) sample was first placed in a stainless‐steel photoreactor with a quartz window and then the photoreactor was purged overnight by a stream of CO_2_ (99.999 %) flowing through a bubbler at 20±2 °C with a flow rate of 1.0 mL min^−1^. The photoreduction tests were performed at 25±2 °C (controlled by a hot plate placed under the photoreactor) and in the presence of a UV lamp as the light source (75 mW cm^−1^, 365 nm) for 8 h. The outlet of the gas was analyzed hourly by an online gas chromatograph (GC, Agilent, Model 7890 B series), which was equipped with a Hayesep Q column (1.5 m, 1/16 inch OD, 1 mm ID), a Molecular Sieve 13X (1.2 m, 1/16 inch OD, 1 mm ID), a thermal conductivity detector (TCD), a nickel‐catalyzed methanizer and a flame‐ionization detector (FID).

The apparent quantum efficiency (AQE) was measured under similar photocatalytic reaction conditions using the same UV lamp (75 mW cm^−1^, 365 nm). The incident flux was determined by Laboratory Spectroradiometer (Apogee Instruments). The AQE values of CH_4_ evolution for CO_2_ photoreduction reaction were calculated according to the following equation:

$$\Phi {\mathrm{CH}}_{4}\left(\%\right)=\frac{8\times \left({\mathrm{number}\;\mathrm{of}\;\mathrm{evolved}\;\mathrm{CH}}_{4}\;\mathrm{molecules}\right)}{\mathrm{number}\;\mathrm{of}\;\mathrm{incident}\;\mathrm{photons}}\times 100\;\%$$

## Conflict of interest

*The authors declare no conflict of interest*.

## Supporting information

As a service to our authors and readers, this journal provides supporting information supplied by the authors. Such materials are peer reviewed and may be re‐organized for online delivery, but are not copy‐edited or typeset. Technical support issues arising from supporting information (other than missing files) should be addressed to the authors.

Supplementary
