# Supplementary material for: Raspberry‐Like Microspheres of Core–Shell Cr2O3@TiO2 Nanoparticles for CO2 Photoreduction
Source: ChemSusChem. 2019 Oct 17;12(24):5246–52. doi: 10.1002/cssc.201901712 (PMC6972636; doi:10.1002/cssc.201901712)
Supplement: Supplementary file 1 — Supplementary [file CSSC-12-5246-s001.pdf]

CHEMISTRY & SUSTAINABILITY
CHEMSUSCHEM
ENERGY & MATERIALS

Supporting Information

Raspberry-Like Microspheres of Core–Shell $Cr_2O_3@TiO_2$ Nanoparticles for CO_2 Photoreduction

Jeannie Z. Y. Tan,*[a] Fang Xia,[b] and M. Mercedes Maroto-Valer[a]

cssc_201901712_sm_miscellaneous_information.pdf

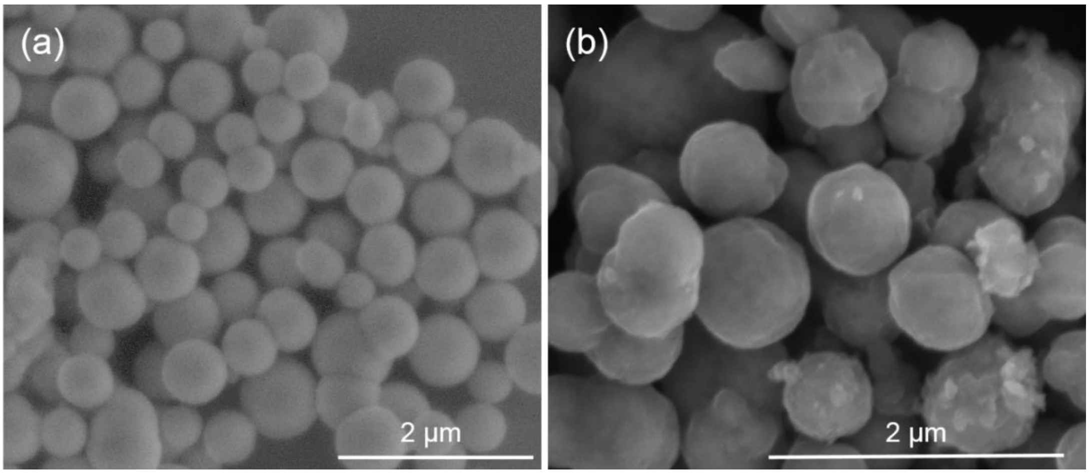

Figure S1. SEM images of Cr_2O_3/SiO_2 (a) and Cr_2O_3/SiO_2/TBT (b).

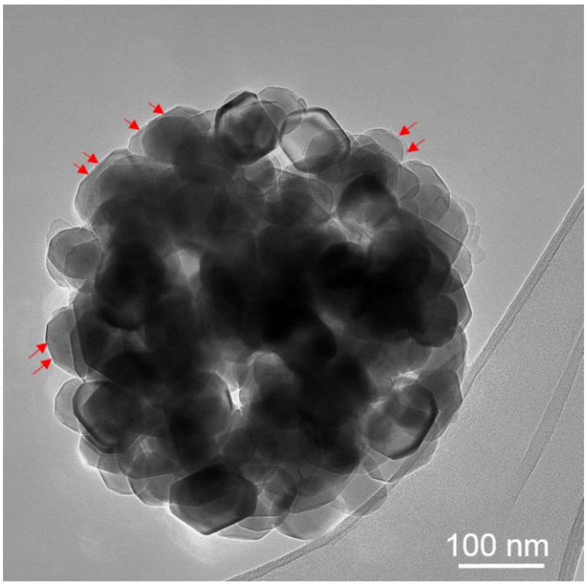

Figure S2. TEM image of Cr_2O_3/titania before calcination treatment. (Red arrow: titania coating)

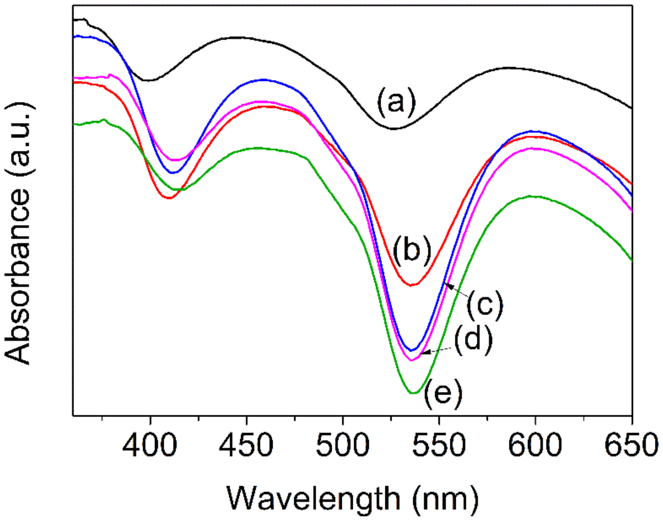

Figure S3. UV-vis spectra of as-prepared Cr_2O_3 (a), 400–Cr_2O_3/TiO_2 (b), 550–Cr_2O_3/TiO_2 (c), 700–Cr_2O_3/TiO_2 (d), 850–Cr_2O_3/TiO_2 (e).

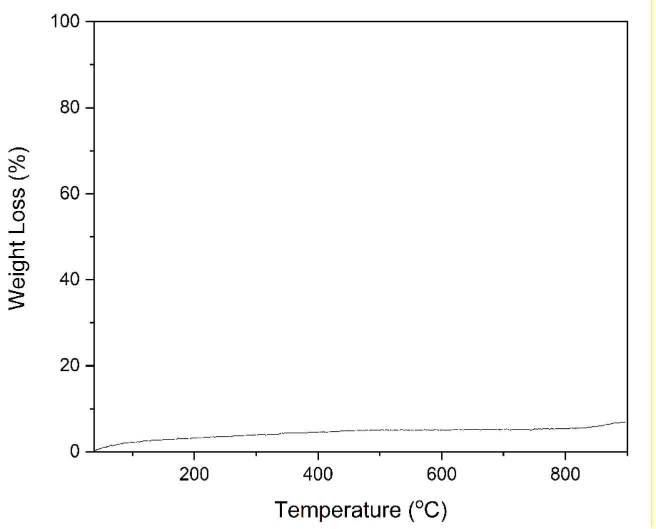

Figure S4. Weight loss against temperature of 400–Cr_2O_3/TiO_2.

Table S1. Cumulative production of CO and CH_4 from CO_2 photoreduction.

Sample name	CO (μmol $g^{-1}_{catalyst}$ h^{-1})	CH_4 (μmol $g^{-1}_{catalyst}$ h^{-1})
Cr_2O_3	0.118	81.730
400-Cr_2O_3/TiO_2	0.135	105.576
550-Cr_2O_3/TiO_2	0.359	147.007
700-Cr_2O_3/TiO_2	0.488	167.689
850-Cr_2O_3/TiO_2	0.258	63.044

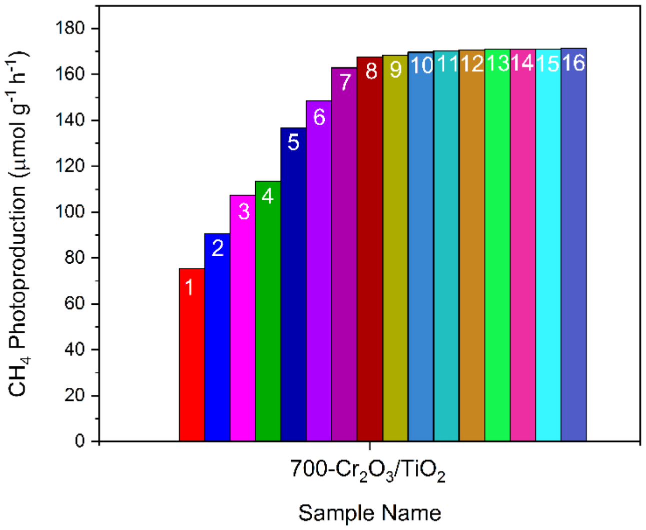

Figure S5. Durability test of 700–Cr_2O_3/TiO_2.

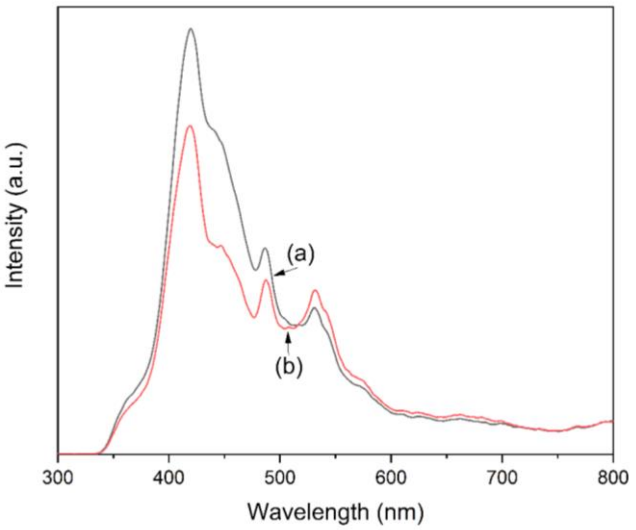

Figure S6. PL spectra of Cr_2O_3 (a) and 700– Cr_2O_3/TiO_2 (b).
